# Technology Readiness Levels for vaccine and drug development in animal health: From discovery to life cycle management

**DOI:** 10.3389/fvets.2022.1016959

**Published:** 2022-12-21

**Authors:** Sven Arnouts, Scott Brown, M. Luisa de Arriba, Michael Donabedian, Johannes Charlier

**Affiliations:** ^1^Provaxs, Department of Translational Physiology, Infectiology and Public Health, Ghent University, Merelbeke, Belgium; ^2^SABAH Consulting, Galesburg, MI, United States; ^3^Laboratorios Syva, León, Spain; ^4^Vétoquinol, Magny-Vernois, France; ^5^AnimalhealthEurope, Brussels, Belgium; ^6^Kreavet, Kruibeke, Belgium

**Keywords:** veterinary, research, funding, therapeutics, biological, pharmaceutical, innovation, public-private partnership

## Abstract

Public research and innovation initiatives in animal health aim to deliver key knowledge, services and products that improve the control of animal infectious diseases and animal welfare to deliver on global challenges including public health threats, environmental concerns and food security. The Technology Readiness Level (TRL) is a popular innovation policy instrument to monitor the maturity of upcoming new technologies in publicly funded research projects. However, while general definition of the 9 levels on the TRL-scale enable uniform discussions of technical maturity across different types of technology, these definitions are very generic which hampers concrete interpretation and application. Here, we aligned innovation pipeline stages as used in the animal health industry for the development of new vaccines or drugs with the TRL scale, resulting in TRL for animal health (TRLAH). This more bespoke scale can help to rationally allocate funding for animal health research from basic to applied research, map innovation processes, monitor progress and develop realistic progress expectations across the time span of a research and innovation project. The TRLAH thus become an interesting instrument to enhance the translation of public research results into industrial and societal innovation and foster public-private partnerships in animal health.

## 1. Introduction

The health of animals, whether livestock, pets or wildlife, is inextricably linked to the wellbeing of people and planetary health. Healthier animals or the systems in which they are kept need fewer natural resources, allowing them to provide more food, labor, fertilizer and companionship while requiring less feed, water and land. Animal health is also a condition for animal welfare. Keeping animals healthy, reduces pollution from livestock production and lowers the risk of transmitting pathogens to humans (1).

Innovations in animal health offer the prospect of a world where the threat of animal disease is much reduced, thanks to stronger immunity, improved prevention strategies, earlier and more specific diagnosis, and new treatments. Moreover, innovations in animal health often spearhead novel developments in human medicine such as in the case of several new vaccine technologies (2). While the gaps in our current knowledge and the need for new therapeutics and vaccines are increasingly being described (3, 4), developing said therapeutics and vaccines seems an increasingly challenging task. Animal health companies nowadays need to invest more time and money to bring new products to the market, potentially explaining the observed dwindling of new animal drug approvals in the EU and USA (5, 6). This is in contrast to the yearly number of human drug approvals, which have increased since the 1970s (6).

Because of the multiple societal benefits of animal health, the public sector invests in research and innovation in the field. For instance, research programme owners and funders who are member of the STAR-IDAZ international research consortium (IRC) on animal health have committed to invest over $2.5 billion in a 5-year period to deliver on improvement and innovation in animal health priorities (7). However, a well-known problem in public funded research and the translation of its results into new products is the so called “valley of death”, meaning that basic research, often conducted at universities and public institutions do not translate enough to industrial applications (8). In order to address this problem in the EU, the European Commission (EC) introduced the use of the Technology Readiness Level (TRL) scale in its key research and innovation funding programme Horizon 2020 and its follow-up Horizon Europe (9). TRLs are used as an innovation policy tool to monitor the maturity of upcoming new technologies at the start and at the end of a project as well as the spread of research and innovation (R&I) investment across different maturity levels (from basic research to applied) (9–11).

The use of the TRL scale as a policy instrument has also been criticized, because (i) uncritical use may skew funding decisions disproportionally toward projects at higher TRL leading to risk averse approaches to economic and societal impact (11) and, (ii) the tool suffers from deficiencies in grasping all essential steps from scientific breakthrough to innovation and reduces the R&I process to a linear pipeline (12). In essence, a more discipline-specific tailoring of the TRL scale has been recommended to increase its value by the above cited organizations.

With the proposal for a European Partnership for Animal Health and Welfare (PAHW) under Horizon Europe, the EC has the ambition to bring together significant resources to deliver in a coordinated way key knowledge, services and products that could improve the control of animal infectious diseases and animal welfare by 2030 (13). The aim of European partnerships is to foster collaboration among participating countries, the private sector, foundations and other stakeholders to deliver on global challenges and modernize industry. As such, they translate broad priorities into concrete roadmaps and activities. To reach this goal, collaboration between academia and other research organizations and industry is required at a certain stage of maturity of new technologies. However, not all industries are familiar with the TRL-concept. In particular, the animal health industry has defined another set of stages of development going from discovery to registration, product launch and pharmacovigilance (14). In order to support the PAHW and other animal health R&I initiatives envisaging public-private collaborations in making optimal use of the available resources and maximally deliver on the needed animal health products, we developed tailored TRL scales adapted to the innovation process for novel vaccines and drugs in animal health. We describe sequentially (i) the general concept of TRL and (ii) the innovation pipeline stages currently applied in the animal health industry and finally (iii) combine the 2 concepts into tailored TRLs for animal health products (TRLAH) that could facilitate collaboration between academia/research organizations and industry as well as the monitoring of public and public-private research initiatives to fill remaining gaps in the animal disease medicine armory.

## 2. The concept of Technology Readiness Levels

**Figure 1:**
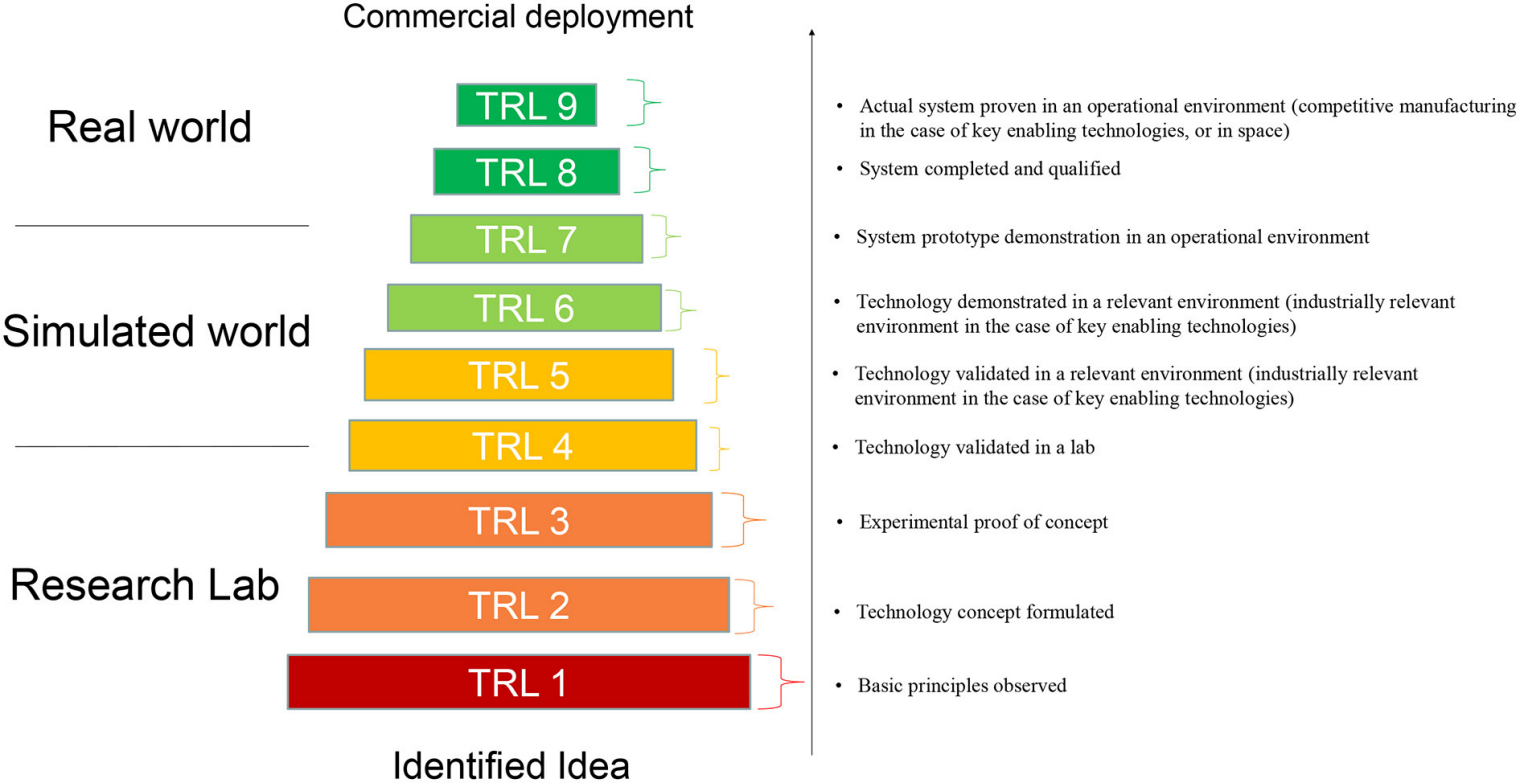
Graphical representation of Technology Readiness Levels (TRLs). The TRLs as defined in the General Annexes of the Horizon Europe work program appear on the right. Graph is based on Maurelli et al. (30).

The TRL scale was originally developed by NASA, where it began as a means of measuring how far a technology was from being deployed in space (9). Later, the scale spread to other governmental departments, and since 2014 has become adopted and is widely used in the EU Horizon 2020 and Horizon Europe and other R&I programmes (15). The TRL scale as used in the EU comprises nine technology readiness levels (TRL 1–TRL 9) (Figure 1). These levels indicate how far a technology is from being fully applied in its intended environment. For example, TRL 2–TRL 4 indicate that the concept is being developed in the laboratory, TRL 5–TRL 7 indicate that the technology is being validated or demonstrated in a relevant environment (piloting), while TRL 8 and TRL 9 imply that the technology is fully implemented, e.g. in a commercial environment.

The TRL scale is a maturity model, and because it is an abstract concept, it can be used to (i) compare different technologies; and to (ii) monitor the progress of one technology over time. TRL thus provide information on the stage of maturity of a technology, not on the process to move from one stage to the other. As Horizon Europe funds several stages of research, the TRL scale is used to indicate boundaries, namely the start and end point of a given technology in a R&I project, and thus the progress that should be obtained during the lifetime of that project. With the TRL scale, the EC gives potential research project applicants an indication of the maturity level of the current research on that topic. This enables applicants and evaluators to align with the expectations of the call topic. The project proposed needs to match with the expected TRL, as it acts as a (soft or hard) eligibility criterion, depending on the type of collaborative project. It helps to assess the specific, and differential, contribution of the EU grant to progress along a linear development scale. Normally, the closer a project is to TRL9, the less likely will it receive any public funding, due to the application of state-aid rules, which prevent from support on commercialization (15).

The high level of abstraction of the TRL scale has a lot of advantages, such as the ability to compare even very diverse technologies. However, it has also been recognized that the TRL concept does not grasp every aspect of the innovation process (9, 11, 12). Other, complementary maturity models and scales have been proposed and used such as the Societal Readiness Level (SRL), Organizational Readiness Level (ORL) or Legal Readiness Level (LRL) (15). In the US, several governmental departments have further adapted the TRL scale to better fit the innovation process in specific sectors such as the adapted TRLs for Medical Countermeasure products by the U.S. Department of Health & Human Services (16), the TRL used by the U.S. Department of Energy (17) or the Biomanufacturing Readiness Levels (BRL) which look at the biopharmaceutical development including diagnostic devices from a strict manufacturing perspective (18).

## 3. Research and development phases for animal medicines

To our knowledge, TRLs for animal medicine development have not yet been described. In contrast, the industry uses since long its own development stages along which critical decisions are taken on further development and commercialization of a particular product. These pipeline stages have been described for animal drugs (19) as well as animal vaccines (14, 20). The whole process is typically divided into 4 phases: Discovery/Research, development, registration and life cycle/product management (Tables 1–**3**). The development phase is further divided in early/preclinical and late/clinical development. Each phase is characterized by a typical set of studies that need to be conducted before the decision is taken to move to the next phase. These studies can be very specific for every product under development. For instance, specific studies performed in antiparasitics discovery have been described by Selzer and Epe (21). The whole process of animal medicine development from initial concept to market typically takes between 5 and 15 years and an investment of up to €150 million (19, 22).

**Table 1:** Technology Readiness Levels for veterinary vaccines.

**Industrial R&D phases**	**TRL**	**Description/definition**
Pre-development phase/discovery/proof of concept	1	Basic research on vaccine target performed, scientific knowledge base reviewed, competitive landscape and market potential assessed
	2	Design and formulation of vaccine candidates (experimental vaccines) and clear product profile defined
	3	Immunogenicity demonstrated in non-target/target species
	4a	Proof of Concept: safety demonstrated in target species
	4b	Proof of Concept: immunogenicity and efficacy (minimum immunizing dose) demonstrated in target species via representative (small scale) animal (challenge) model
	4c	Vaccine candidate and formulation selected
Development phase: early/preclinical development	5	Animal safety demonstrated in target species; For live genetically modified organisms, demonstrate safety in non-target species; evaluation of user safety, environmental safety and residues
	6	Efficacy demonstrated in a representative and validated target animal challenge model (if available)
Development phase: late/clinical development	7	Safety and/or efficacy evaluated under relevant (field) conditions
Registration phase	8	Final vaccine defined and regulatory dossier completed, ready for Market Authorization Application, evaluation, response to questions and approval
Life cycle	9	Vaccine marketed and evaluated in the field (pharmacovigilance)

In order to assist public-private collaboration in animal vaccine and drug developments, a number of process maps have been developed. The vaccine development process was documented in a Veterinary Vaccine Development Process Map which covers the process from the generation of a Target Product Profile (TPP) through discovery and feasibility to product development and registration (14). It is primarily used to facilitate the development of new vaccines for academics who are less familiar with the details of a commercial vaccine development process and the complexity of regulatory requirements. In addition, the STAR-IDAZ IRC constructed generic research roadmaps for the development of candidate vaccines, therapeutics as well as diagnostics and control strategies (23). These roadmaps describe the building blocks and for each the key research questions, dependencies, challenges and possible solution routes to identify the research needed to achieve a specific TPP. The roadmaps are completed independently for specific diseases and complemented by information on ongoing research. As such it is used by the animal health research community including funding organizations to identify research gaps that need to be addressed (23).

## 4. TRLs for animal medicines

Below, we adapt and specify the TRL scale as applied in the EU innovation funding schemes to match the different TRL-scales with the pipeline stages in animal vaccine and drug development as they are used by the animal health industry. Across the TRL scales for vaccines and drugs, TRL 1–4 cover the discovery phase, TRL 5–7 the development phase, TRL 8 the registration phase and TRL 9 the marketing and product support phase. Next to the definition of the different scales of the TRLAH we provide a brief description of the studies that should be performed or the material that should be provided to reach that scale. For more detailed information on this matter we refer to guidelines of the International Cooperation on Harmonization of Technical Requirements for Registration of Veterinary Medicinal Products (VICH). VICH guidelines are developed to harmonize registration guidelines for safety, efficacy and quality assessments between the EU, US, and Japan. However, these guidelines do not exist for every aspect of registration yet. Associated activities from the manufacturing that are linked to some TRL are also described.

### 4.1. Vaccines

The vaccine TRL scale (Table 1) describes increasing levels of maturity from basic research on a vaccine target, the competitive landscape and the market potential (TRL1) to a marketed vaccine (TRL 9). The development phase starts at TRL 5, where safety studies are conducted under controlled laboratory conditions. These studies include Good Laboratory Practice (GLP) single dose, repeated dose and overdose studies. Efficacy studies under controlled laboratory conditions are conducted at TRL 6. These studies are also named pre-clinical efficacy studies. Here, vaccination and challenge trials are conducted for dose determination and confirmation and/or to assess the onset and duration of immunity. Clinical trials start at TRL 7 where safety and efficacy trials are performed at large scale under field conditions.

Each of these stages in the development process requires the production of different prototype vaccines from the manufacturing side. From TRL 1–4 an experimental vaccine at lab-scale is used. At the end of this phase the industrial production process is defined and necessary control methods (in-process controls and final product controls) are established. For TRL 5 the production of a pilot batch of vaccine (with maximum potency) is required for safety studies as well as the Master Seed material. The pilot batch is manufactured by a procedure fully representative of and simulating the procedure at commercial scale. The methods of cell expansion, harvest, product purification and formulation should be identical as for commercial production and tested using established in-process and final product control methods according to VICH guidelines. From TRL 6 process validation and manufacturing according Good Manufacturing Practice (GMP) regulations is required, although differences in the requirements exist between different countries. A minimum potency (lowest dose) production batch is typically used for efficacy studies. The active ingredient and final formulation are scaled up to production scale. Validation requires at least 3 production batches tested using validated in-process and final product control methods. This leads to the production batch: it is manufactured in the intended production facility by the method described in the application for market authorization. It includes the establishment of stability data of both the active ingredient and the final product to define the intended shelf life.

### 4.2. Drugs for food animals

The TRL scale for drugs in food animals (Table 2) describes increasing levels of maturity from basic research on the target, the competitive landscape and the market potential (TRL1) to a marketed drug or treatment (TRL 9). Safety and efficacy studies are initiated at TRL 5 and 6, respectively, whereas multi-location field safety and efficacy studies with the final formulation for the commercial drug are performed at TRL 7.

**Table 2:** Technology Readiness Levels for development of drugs for food animals.

**Industrial R&D phases**	**TRL**	**Description/definition**
Pre-development phase/discovery/proof of concept	1	Basic target observed, scientific knowledge base reviewed, competitive landscape and market potential assessed
	2	Drug/treatment concept formulated (development of hypothesis and experimental designs) and clear product profile defined
	3a	Experimental proof of concept demonstrated in a limited number of *in vitro* models (establish IC50 or other relevant potency threshold using biomarkers); *in vitro* toxicology studies including mutagenicity performed
	3b	Experimental toleration study performed in target species; toxicology studies in rodent, non-rodent and target species (up to 30-day); Radiosynthesis (metabolism and residue studies) initiated
	3c	Experimental proof of concept demonstrated in a limited number of *in vivo* models including pharmacokinetic-pharmacodynamic modeling; target animal metabolism studies and target tissue for residues determined; residue methods development commences
	4	Efficacy demonstrated in representative (small scale) naturally occurring disease model to establish dosage regimen; 90-day toxicology studies initiated
Development phase: early/preclinical development	5	Assessment of animal safety and initiation of environmental safety, user safety, and (if needed) microbiological safety assessments; carcinogenicity studies initiated if needed
	6	Efficacy and safety demonstrated at large scale (sufficient statistical power) in a representative animal model; toxicology studies completed; preliminary residue methods compared with total residues and marker residue identified; preliminary Allowable Daily Intake calculated
Development phase: late/clinical development	7	Final formulation for commercial drug/treatment multi-location field efficacy and safety study; residue decline studies conducted, residue methods finalized; withdrawal period proposed at final dosage regimen, and preliminary withdrawal period (and milk discard, if applicable) calculated
Registration phase	8	Final drug/treatment defined and ready for Market Authorisation Application, evaluation, response to questions, and approval
Life cycle	9	Drug/treatment marketed and evaluated in the field (pharmacovigilance)

From the manufacturing side, TRL 1–4 are based on the experimental formulation at lab-scale, leading to the definition of an industrial production process and the establishment of necessary control methods (in-process and final product controls). Impurities are identified and characterized. From TRL 5 a Pilot batch of Final Drug Product (FDP) is required: a batch of FDP manufactured by a procedure fully representative of and simulating the procedure at commercial scale. The production process and formulation are optimized. The methods of synthesis or cell expansion, harvest or yield, product purification and formulation should be identical as for commercial production and tested using established in-process and final product control methods. Impurities are qualified and/or tested for safety concerns. Preliminary stability studies are done to identify and characterize degradants and degradation pathways. Safety assessment on degradants is performed as needed per VICH guidelines. As for vaccines, from TRL 6 onwards, the production process needs to be validated and manufacturing needs to occur according to GMP rules. The active ingredient and final formulation are scaled up to production scale and the validation requires at least 3 production batches using manufacturer validated in-process and final product control methods. This leads to the production batch: a batch of FDP manufactured in the intended production facility by the method described in the application for market authorization. It includes the requirement to establish stability data of both active ingredient and final product to define the intended shelf life.

### 4.3. Drugs for companion animals

The TRL scale for drugs for companion animals (Table 3) follows the same logic as the one for drugs for food animals but is more straightforward because it requires no or fewer studies related to consumer safety and impact on the environment. The requirements on the manufacturing side are the same as described above for drugs in food animals.

**Table 3:** Technology Readiness Levels for development of drugs for companion animals.

**Industrial R&D phases**	**TRL**	**Description/definition**
Pre-development phase/discovery/proof of concept	1	Basic target observed, scientific knowledge base reviewed, competitive landscape and market potential assessed
	2	Drug/treatment concept formulated (development of hypothesis and experimental designs) and clear product profile defined
	3a	Experimental proof of concept demonstrated in a limited number of *in vitro* models (establish IC50 or other relevant potency threshold using biomarkers)
	3b	Toxicology studies performed in target species
	3c	Experimental proof of concept demonstrated in a limited number of *in vivo* models including pharmacokinetic-pharmacodynamic modeling
	4	Efficacy demonstrated in representative (small scale) naturally occurring disease model to establish dosage regimen
Development phase: early/preclinical development	5	Animal safety assessed and initiation of environmental safety, user safety, and (if needed) microbiological safety assessments
	6	Efficacy and safety demonstrated at large scale (sufficient statistical power) in a representative animal model
Development phase: late/clinical development	7	Final formulation for commercial drug/treatment multi-location field efficacy and safety study
Registration phase	8	Final drug/treatment defined and ready for Market Authorization Application, evaluation, response to questions, and approval
Life cycle	9	Drug/treatment marketed and evaluated in the field (pharmacovigilance)

## 5. Discussion

The One Health and One Welfare concepts are increasingly recognized and adopted in health policies around the world (24, 25). Implementation of these concepts requires the generation of new knowledge as well as new, improved and accessible control tools such as vaccines and other medicines to secure animal, public and environmental health. The Tripartite (FAO, WOAH, and WHO) considers use of vaccines as a key strategy to reduce the need for antibiotic usage in agricultural production, animals and humans (26). However, other tools and knowledge are needed as well and in the animal health domain, these are described in public databases such as DISCONTOOLS (www.discontools.eu) or reports from various stakeholders or disease-specific research alliances [e.g., (27)]. Downstream initiatives such as STAR-IDAZ IRC then attempt to coordinate globally public funded research to deliver on the identified needs (23).

The animal health industry is a global, competitive market where, as in many other sectors, it is increasingly difficult to innovate via in-house research and processes. Since long, the animal health industry has innovated in close interaction with academia and public research institutions. However, the translation of public research results into industrial and societal innovation has often been hindered by lack of public funding for proof-of-concept studies and by different interpretations of the level of maturity of the technology between academia and industry.

Nowadays, more public funding opportunities are offered to stimulate collaboration between academia and industry, both at the level of the EU as by national and regional funders. Examples funded by the EC are Research and Innovation Actions, the EU partnerships and funding opportunities via the European Innovation Council. An example at the regional level in Flanders are several products of VLAIO (Flemish Institute for Innovation and Entrepreneurship) that funds collaborative R&D-activities between universities and industry where either the university or the industry entity can be the lead applicant. In addition, an Industrial Research Fund (known as IOF) supports proof of concept studies and technology transfer activities at the five universities in Flanders. Another interesting initiative to bridge the gap between academia and industry is the innovation office of EMA (European Medicines Agency) and its national agencies. These offices have been launched to facilitate and support innovation in pharmaceutical R&D and the communication with the innovators. They are intended to serve as the central access point to the existing scientific and regulatory expertise of the agencies both for human and animal medicines and therapies. They are accessible for pharmaceutical companies, small and medium-sized enterprises (SMEs), academic research centers, spin-offs, academic hospitals and individuals who are actively involved in pharmaceutical innovation in general and particularly in R&D of new medicines and therapies. On the other hand, EMA published a list on regulatory science needs as also from the regulatory side research is needed to close gaps and improve medicine development and evaluation to enable access to innovative medicines (28). By engaging in these regulatory science research needs initiative, researchers and funders will be able to see their findings translated into regulatory practice, medicines development and public health. Also for this initiative clear definitions of TRL will be useful to communicate on the research needs and objectives to funders and researchers.

The candidate PAHW has the ambition to encourage public-private partnerships (PPP) to turn novel research results and technologies into actual products that support the economy while delivering societal impacts (13). The form of collaboration in PPPs and the role of the public/academic or industrial partner strongly depend on the TRL (29). The developed TRLAH make it clear that basic research (TRL 1–4) can be performed independently by either public institutions like academia or industry. However, from TRL 5 onwards, the associated regulatory environment is increasingly demanding. Expert knowledge on the regulatory requirements of materials used and on study design (according to pharmacopeia and monographs) is then required next to expert knowledge on the novel technology and can prevent that expensive research trials need to be repeated. PPPs that aim at reaching the higher TRL to increase the impact of their investment in research and development should enable both public and private partners to play their specific roles as they move through the different TRL.

Outside of large PPP, the TRLAH can be useful to facilitate the transfer of new technologies from research institutes/academia to private companies, either to animal health companies or to new spin-offs/start-ups. In that case the TRL can be used to clearly define the maturity of the technology which is an essential basis to start its valuation. The latter being a critical element to negotiate the conditions of the license to the animal health company or to the start-up/investors.

Finally, innovation in animal medicines is an ongoing process with completely new products on the horizon such as nanoparticles, nanobodies or functional nutritional products. For such products industrial stage gates are not yet well developed and a clear regulatory framework is often lacking. Such novel products may require further adaptation and refining of the proposed TRLAH in a continuous co-design approach between industry, regulators and innovation providers.

## Author contributions

SA and JC developed the concept and wrote the paper. SA, SB, MA, and MD developed the TRL scales for animal medicines. All authors have read and approved the manuscript.
